# Differential Gene Expression Patterns in Peach Roots under Non-Uniform Soil Conditions in Response to Organic Matter

**DOI:** 10.3390/genes15010070

**Published:** 2024-01-04

**Authors:** Brian T. Lawrence, Alejandro Calle, Christopher A. Saski, Juan Carlos Melgar

**Affiliations:** 1 Department of Plant and Environmental Sciences, Clemson University, 105 Collings Street, Clemson, SC 29634, USA; 2 Horticulture Section, School of Integrative Plant Science, Cornell University, Geneva, NY 14456, USA; 3 Institut de Recerca i Tecnologia Agroalimentàries (IRTA), Fruitcentre, PCiTAL, Gardeny Park, Fruitcentre Building, 25003 Lleida, Spain

**Keywords:** compost, soil biology, soil carbon, transcriptome, *Prunus persica*

## Abstract

Organic matter (OM) amendments are often encouraged in sustainable agriculture programs but can create heterogeneous soil environments when applied to perennial crops such as peaches (*Prunus persica* (L.) Batsch). To better understand the responses of peach roots to non-uniform soil conditions, transcriptomic analysis was performed in a split-root study using uniform soil (the same soil type for all roots) or non-uniform soil (different soil types for each half of the root system) from either (1) autoclaved sand (S), (2) autoclaved sand with autoclaved compost (A), or (3) autoclaved sand with compost which included inherent biological soil life (B). Each uniform soil type (S, A, and B) was grouped and compared by uniform and non-uniform soil comparisons for a total of nine treatments. Comparisons revealed peach roots had differentially expressed genes (DEGs) and gene ontology terms between soil groups, with the S and B groups having a range of 106–411 DEGs and the A group having a range of 19–94 DEGs. Additionally, six modules were identified and correlated (*p* > 0.69) for six of the nine treatment combinations. This study broadly highlights the complexity of how OM and biological life in the rhizosphere interact with immediate and distant roots and sheds light on how non-homogenous soil conditions can influence peach root gene expression.

## 1. Introduction

One of the sustainable development goals outlined by the United Nations (UN) places emphasis on achieving sustainable food production by increasing yields and rehabilitating degraded lands using organic fertilizers [1]. In perennial fruit tree management, the benefits of adding organic matter (OM) amendments to improve soil quality and provide mineral nutrients have been documented [2,3] as incorporations can enhance soil aggregation, improve nutrient cycling and nutrient availability, support beneficial soil biology, and promote healthy, productive trees. However, the precise impact of OM on the gene expression of fruit tree roots growing in soil with increased OM is largely unexplored. OM plays an important role in soil fertility, as its decomposition over time can release essential macro- and micronutrients required for plant growth. The release of these nutrients and increasing the humic acid fraction can have a significant impact on the composition and activity of microorganisms [4,5]. Populations of bacterial and fungal communities within the rhizosphere, such as mycorrhizae, can also improve nutrient and water acquisition of plants [6,7]. Microorganism–root associations are initiated and regulated by the composition of root exudates, which are influenced by abiotic or biotic factors within soil conditions [8]. In addition to localized responses within root tissue, increases in root associations with microorganisms in the soil may result in differentially expressed genes (DEGs) in other portions of the plant, including the leaves and fruit [9,10], while compost extracts can induce systemic resistance [11]. Abiotic factors including water availability [12], humic substances [13], and inorganic nutrients [14,15] have already been shown to alter root gene expression but little work has explored how non-uniform soil conditions from an increase in OM can influence the plant system.

While adding OM is not a common practice in commercial fruit tree orchards, increasing soil OM can potentially improve the orchard ecosystem and economy [16,17]. However, in contrast to mineral fertilizers, which can be spread evenly, adding organic amendments to increase OM can often result in heterogeneous soil environments for growing trees. Whether these variations in soil conditions, i.e., an increase in OM in proximity to several roots, substantially change gene expression in other roots of the same plant has not been studied but could help us optimize factors such as the timing, use, and location of OM application in modern tree management and better understand how factors such as nutrient uptake, tolerances to stresses, or interactions with the rhizosphere impact tree performance. Although tree root responses and systemic signaling to heterogeneous soil conditions are inherently complex, current transcriptomic tools allow roots of the same plant growing in different soil conditions to be compared. In the current study, the peach tree is used as a model since the genome has been available to the scientific community for over a decade and has a fairly simple genome compared to other fruit tree species [18]. Considering the adoption of organic fertilizers promoted by the UN and the potential increase in OM content within orchard settings, this study employed a split-root design of clonal peach trees to explore the DEGs of roots growing in non-uniform soil conditions. The purpose was to explore how increasing OM, with or without soil microbiology (soil life) present, would change the gene expression of roots within adjacent soil.

## 2. Materials and Methods

### 2.1. Design and Treatments

The experiment was performed in a greenhouse at Clemson University, South Carolina, USA (34°67′25.41″ N, 82°83′57.27″ W). The greenhouse had an average temperature of 25.4 °C ± 3.1 °C (mean ± standard error) and a relative humidity between 42% and 82% receiving natural light and supplemental light for a day length between 6 AM and 10 PM. A total of 24 ‘Guardian^®^’ peach rootstocks (*Prunus persica* (L.) Batsch, ‘Guardian’) were propagated using tissue culture one year prior to the study [19]. After a single year of growth, the propagated trees were small and ranged in size from 5 cm to 10 cm tall before being initially potted in 6.8 cm × 22.8 cm × 6.8 cm tree pots (Anderson Pots, Portland, OR, USA) with 50% Fafard 3B potting mix containing Canadian sphagnum peat moss, bark, perlite, vermiculite, dolomitic limestone (Sun Gro Horticulture, Agawam, MA, USA), and 50% sand for approximately 2 months to allow for root development. Before transplanting from these initial pots, the following soil types were prepared: autoclaved sand (All Purpose Sand, American Countryside, Muscle Shoals, AL, USA) as a control (S), autoclaved sand and autoclaved compost (A), and autoclaved sand and non-autoclaved compost with indigenous biological soil life, including microorganisms (B). The compost used was acquired from the Cherry Crossing Research Center (Clemson University Composting Facility) and was a stabilized mixture of yard debris and food waste from the university cafeterias and sifted to ≤2 mm size. Analysis of the compost including ICP analysis of element composition, C:N ratio, OM (loss on ignition), electrical conductivity (EC), and pH was performed by the Clemson University Agricultural Service Laboratory in Clemson, SC, USA, and can be found in Table 1.

The amount of compost applied to each pot was 19.9 g dry weight, the equivalent of a 44.8 Mg ha^−1^ (20 tons acre^−1^) rate based on the pot depth (22.8 cm deep). Each treatment was homogenized in a cement mixer (and then autoclaved in the cases of soil types A and S) before being placed into tree pots (5.4 cm^2^ × 22.8 cm). On the same day when the tree pots were filled with the three soil types, trees were removed from the initial pots, the previous soil was removed completely by washing the roots, and trees were planted into the experimental tree pots using a split-root system so that the roots of each tree were divided approximately in half, and adjacent halves were planted into contiguous tree pots, enabling the shoot of the tree to rest between where the two pots came together.

The experiment had a total of 24 trees which were divided between two pots, and a letter was assigned to each pot based on the soil type as follows: AA, BB, SS, AB, AS, and BS. Each of these combinations was replicated 4 times. Since the aim of the study was to determine how roots in a specific soil (pot) respond to the influence of the adjacent soil, whenever a pot was selected for root sampling, the other one was considered the adjacent soil. Thus, treatment codes were given to each sample depending on the soil it was taken from (first letter) and the adjacent pot (second letter); the contribution of the adjacent soil was categorized by factor(s) in comparison to the soil in which roots were sampled: for instance, A as an adjacent soil next to S (SA) was a factor of OM, or B as an adjacent soil next to A (AB) was a factor of soil life. The AB, AS, and BS treatments were mirrored during root harvest and additionally served as the following treatments: BA, SA, and SB. Thus, nine treatments were established based on where the roots were sampled and are displayed in Table 2.

After planting into the experimental tree pots, each tree was pruned to have between 7 and 10 leaves, and a single square of white weed cloth was placed on the surface of each pot to avoid soil displacement or loss during watering. The pots were initially watered with tap water until they dripped, then moisture was maintained using 70 mL of a Hoagland nutrient solution (5 mM KNO_3_, 5 mM Ca(NO_3_)_2_, 1 mM KH_2_PO_4_, 2 mM MgSO_4_, 46.3 µM H_3_BO_3_, 9.2 µM MnCl_2_·4H_2_O, 0.8 µM ZnSO_4_·7H_2_O, 0.3 µM CuSO_4_·5H_2_O, 2.8 µM H_2_MoO_4_, and 10 µM Fe-EDTA) every 2–3 days for 8 weeks after transplanting. Following 8 weeks, trees were measured for total shoot length (main shoot and side shoots) before two of the three replicates from each combination were destructively harvested with each root half cut at the soil line. All soil was removed by submersing the root system under running distilled water, then three fibrous root subsamples were cut and wrapped in aluminum foil from each root half of each harvested tree (approximately 0.3 g fresh weight for each sample and treatment) and immediately placed in liquid nitrogen. Therefore, each biological replicate had 3 subsamples of root tissue and the time from cutting the roots at the soil line to placing each root sample in liquid nitrogen was less than one minute. All samples were then stored at −80 °C until RNA extraction.

The roots from the remaining three uniform treatment pots (one pot each of AA, BB, and SS) were washed completely of soil media and stored within plastic bags at 4 °C for 24 h before they were scanned using an Epson Perfection V600 6400 dpi resolution image scanner (Epson, Long Beach, CA, USA). Scanned images were then analyzed using the WinRHIZO Pro image analysis system (Regent Instruments, Inc., Quebec City, QC, Canada) for total root surface area, length, volume, average root diameter, and the number of root tips, forks (where a root visibly separated into two parts), and crossings (where roots overlapped one another).

### 2.2. RNA Extraction, Sequencing, and Data Analysis

Root subsamples from each biological replicate were combined and ground to a powder using a mortar and pestle while frozen, keeping 100 mg of powder as the amount for later RNA extraction in 2 mL tubes. Processing six samples at a time, 20 µL 2-mercaptoethanol was added to each tube before adding 1 mL of extraction buffer (2% CTAB (*w*/*v*), 20 mM EDTA, 1.4 M NaCl, 1% PVP (polyvinylpyrrolidone Mr 40,000), 0.1% DEPC, 100 mM Tris (pH = 7.6)) and 0.8 mL chloroform, then the tubes were immediately transferred to a 65 °C hot plate for 20 min. These samples were then centrifuged for 30 min, and 0.8 mL of supernatant was removed and loaded into a separate 2 mL tube. A 3M LiCl solution was added to the supernatant and tubes were inverted carefully several times before precipitating nucleic acids at −20 °C for >2 h. Following precipitation, tubes were centrifuged for 30 min, and the supernatant was completely removed. After a single rinse and removal of 70% EtOH, samples were resuspended in a 40 µL mixture of DNase, buffer, and DEPC water. Sample RNA was confirmed using gel electrophoresis before sequencing.

Total RNA was quantified using a double-stranded dye-binding assay on the qubit (ThermoFisher Scientific, Waltham, MA, USA). Each RNA sample was normalized to a standard input concentration (1 μg of total RNA), and an Illumina-compatible sequencing library was prepared with the NEBNext Ultra II RNA Library Prep Kit for Illumina (New England Biolabs, Ipswich, MA, USA) following the recommended procedures of the manufacturer. The resulting cDNA libraries were size validated on an Agilent 2100 Bioanalyzer System to ensure proper fragment distribution (∼260 bp) and effective removal of adapter dimers. Sequence libraries were multiplexed and paired-end reads for each sample (2 × 150 bp) were collected on an Illumina NovaSeq 6000 S4 flow cell to an approximate depth of 20 million read pairs for each sample. 

Raw data (raw transcriptomic sequences) were preprocessed for adapters and low-quality read removal using Trimmomatic software (v.0.39) [20]. Trimmed sequence reads were aligned to the *P. persica* reference genome (v.2.0.a1) [21,22] with the latest release of the Bowtie2 short-read aligner [23]. Alignment files were formatted and indexed with Samtools v.1.12 [24]. Transcript quantification was performed with RSEM v.1.3.3 [25]. Raw counts and trimmed mean of the M-values (TMM) [26] were obtained and put in tabular format as a gene expression matrix (GEM). Differential gene expression profiles were determined by normalizing the raw count GEM in edgeR v.3.16 [27] and performing all possible pairwise comparisons. Genes considered significantly differentially expressed for post hoc analysis had a *p*-value ≤ 0.001, a false discovery rate (FDR) of ≤0.05, and a logarithm of fold change (LogFC) of ≥|2|. Comparisons of DEGs were made between each uniform soil condition and the respective groups (A, B, or S) based on where root samples were collected. Graphical visualization of gene expression was conducted using the open-access platform heatmapper (www.heatmapper.ca, accessed on 28 June 2023; [28]).

### 2.3. Gene Ontology (GO)

GO enrichment analyses were obtained from the online database http://www.geneontology.org, accessed on 20 April 2023 [29]. A false discovery rate (FDR) of 0.05 was employed to conduct enrichment analyses on GO terms related to biological processes, cellular components, and molecular function.

### 2.4. Co-Expression Network Analysis

All DEGs found in this dataset were considered for weighted gene co-expression analysis. The co-expression network was constructed using the WGCNA v 1.70–3 package implemented in R [30] with a minimum module size of 10 genes and 0.25 as the merge cut value. The correlation between modules and treatments was determined using Pearson’s correlation coefficient at a significance level of *p* < 0.05.

### 2.5. Statistical Analysis

Analysis of variance (ANOVA) was used to determine if there were differences in total shoot length between treatments during the time of root harvest (α = 0.05) using JMP (Version 14.1.0; SAS Institute, Cary, NC, USA).

## 3. Results and Discussion

### 3.1. Plant Growth Prior to Root Sampling

**Figure 1:**
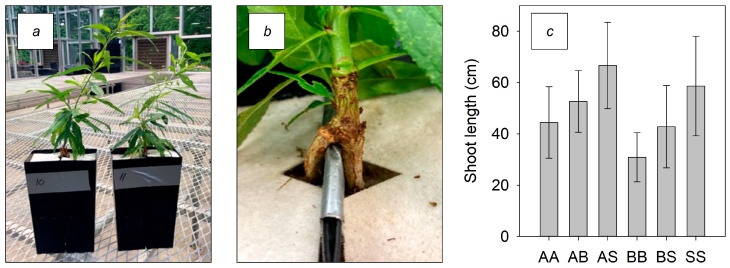
All treatment combinations had successful peach shoot growth after treatments were established between the two pots (**a**), with roots growing into either side of the pots (**b**), and similar shoot length (**c**), (n = 3), between the treatments AA (autoclaved sand plus autoclaved compost in both pots), AB (autoclaved sand plus compost next to autoclaved sand plus biologically active compost), AS (autoclaved sand plus compost next to autoclaved sand), BB (autoclaved sand plus biologically active compost in both pots), BS (autoclaved sand plus biologically active compost next to autoclaved sand), and SS (autoclaved sand in both pots).

All trees grew new shoots after treatments began (Figure 1a) and roots successfully grew within both sides of the contiguous pots (Figure 1b). The average shoot length was similar at the time of root extraction between the treatments (Figure 1c).

The single measurements of root growth made from the remaining trees prevented statistical comparisons, but both AA and SS roots were numerically longer with more root tips, forks, and crossings than BB (Table 3). However, the root diameter and volume of BB were numerically larger than AA and SS. Previous work has shown that adding compost can increase peach root growth in comparison to mineral fertilizer alone because of improved physical soil characteristics and stimulation from humic acids [31]; however, the current study appeared to have similar growth between the SS and AA soil treatments. Associations with soil life, including plant growth-promoting rhizobacteria or arbuscular mycorrhizae fungi (AMF), can improve plant growth [32], but associations with AMF have been shown to occasionally reduce growth, as observed after inoculated vermicompost was applied to sorghum [33]. Although additional measurements are needed, it appears that when growing in biologically active compost (BB), the fine peach roots may become larger in diameter but much shorter in overall length, reducing the number of root tips and forks. While morphological differences occurred, more pertinent to the current study were differences in gene expression between the treatments.

### 3.2. Differential Gene Expression, Relative Expression, and Ontology

Treatment comparisons of DEGs showed differences between the uniform soil conditions and the non-uniform conditions created from the possible combinations of the A, B, and S soil groups (Table 4). The total number of DEGs in treatments with uniform soil conditions increased when OM was a factor, as the least number of DEGs were found between the factor of soil life (AA vs. BB) and the greatest number between both OM and soil life factors (SS vs. BB). The least amount of DEGs was found with the factor of soil life, but it is not possible to know the number of associations made between the plant roots within the biologically active compost soil (B). If the process of associating with soil life increased the number of DEGs, it is possible that the nutrient solution supplied sufficient or elevated inorganic phosphate to the soil, which may have reduced signaling from plant roots, such as the production of strigolactones to form AMF associations [34].

Non-uniform soil conditions showed an influence of the adjacent soil factor depending on the treatment combination. Together, the S group had the highest collective number of DEGs identified (832), followed by the B group (677) and A group (196). The control soil comparisons (S group) showed similar DEGs between the single factor of OM (SA vs. SS) or soil life (SA vs. SB), while the highest number of DEGs was identified when the two factors were combined (SB vs. SS). When comparing SA vs. SB, the SA treatment had nearly three times as many upregulated genes compared to downregulated. GO analysis showed enriched biological process categories from this comparison being mainly related to metal ion (GO:0030001) and transmembrane transport (GO:0055085) (Supplemental S1). Upregulation of these key biological processes may have numerous roles such as nutrient availability and uptake, metal ion homeostasis, and stress response. For example, OM can chelate metal ions (manganese, zinc, copper, etc.), making them more available to plants [35,36]. Metal ion homeostasis is also important for proper plant physiological processes. The activation of transmembrane genes could be interpreted as a signature of the uptake, transport, and localization of metal ions in various cellular compartments [37]. Furthermore, the presence of OM may influence the soil’s redox potential (and possibly pH), which can affect the bioavailability of metal ions and lead to the activation of stress-responsive genes, helping them adapt to these altered conditions.

Comparing SB vs. SS, twice as many DEGs were downregulated compared to upregulated. Upregulated genes were associated with metabolic processes (GO:0008152) and downregulated genes with response to hormones (GO:0009725) (Supplemental S1). In the autoclaved OM soil (A group), the highest number of DEGs was found between single factor differences (AS vs. AA and AB vs. AA) and the lowest when both OM and soil life factors were compared (AS vs. AB). In these comparisons, GO analyses reported genes associated with diverse biological processes, molecular functions, and cellular components, with the larger number of upregulated genes in the AS vs. AA comparison being associated with metabolic processes (GO:0008152) (Supplemental S1), as reported for upregulated genes in the SB vs. SS comparison. The soil with increased OM and biological life present (B group) showed fewer DEGs when comparing the factor of soil life (BA vs. BB) and the two factors of OM and soil life (BS vs. BB) compared to the single factor of OM (BS vs. BA). Both the BA and BS soil had more than twice the number of genes upregulated than downregulated in comparison to BB. Detailed GO analysis table reports for each treatment comparison are shown in Supplemental S1.

### 3.3. Clustering of DEGs by Modules

**Figure 2:**
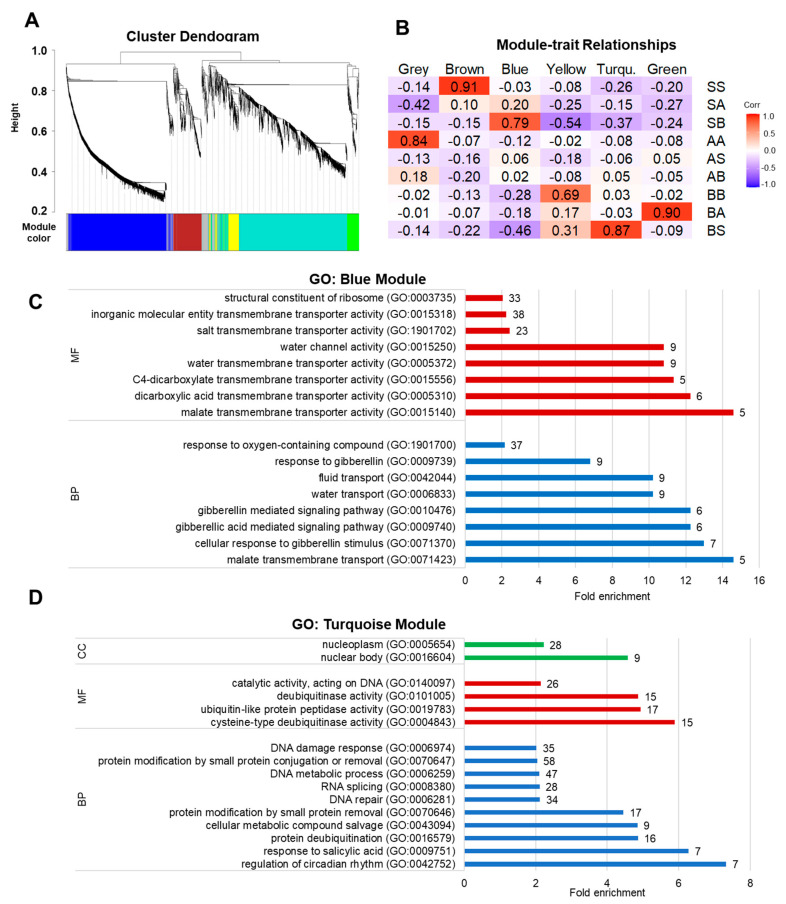
Co-expression network of all differentially expressed genes. (**A**) Dendrogram of gene clustering and modules of coexpressed genes with similar expression patterns clustered by color. (**B**) Correlations (*p*-values) between treatment and modules. Positive and negative correlations are indicated by red and blue coloration, respectively. (**C**,**D**) Enrichments in gene ontology (GO) terms for biological processes (BP), molecular function (MF), and cellular components (CC) for the blue (**C**) and turquoise (**D**) modules. Fold change enrichment and number of genes in each GO term are indicated.

All DEGs were also examined using hierarchal clustering (Figure 2A), which resulted in six significant modules of genes displaying similar expression profiles (Figure 2B). The number of genes in each module ranged from 155 (green module) to 1498 (turquoise module). While the SS correlated with the brown module (*p* = 0.91), AA with the grey module (*p* = 0.84), and BB with the yellow module (*p* = 0.69), neither of these modules resulted in significant GO terms. Non-uniform soil conditions either strongly correlated with other modules, such as SB with blue (*p* = 0.75), BA with green (*p* = 0.90), and BS with turquoise (*p* = 0.87), or showed moderate correlation, such as treatments SA, AS, and AB (Figure 2B). The two modules that had significant GO terms were the blue (Figure 2C) and turquoise modules (Figure 2D), corresponding to the SB and BS treatments, respectively. Significant GO terms for the blue module (correlated to SB treatment; *P* = 0.75) included molecular function (MF) related to water channels (GO:0015250), transmembrane transport of water (GO:005372), along with other transmembrane transport terms, as well as biological processes (BP) associated with fluid (GO:0042044), and water transport (GO:0006833). The SB treatment samples were grown in inert sand, with half of the plant in soil with OM and soil life. Plants were regularly watered, but sandy soils without elevated OM may have been subject to less soil moisture in comparison to soils with OM and transport of water from these roots may have occurred [38]. In a study with a chrysanthemum relative, *Opisthopappus taihangensis*, genes conferring drought tolerance, such as long-chain acyl-CoA synthetase, were found to be upregulated [39]. This aligns with the results found in our analysis, where two of these genes (Prupe.1G167500 and Prupe.1G168200; annotated as long-chain acyl-CoA synthetases) were upregulated when growing in the inert sand, which could be linked to a response to drought stress. The response of SB to gibberellin (GO:009739), pathways of gibberellin (GO:0010476), or gibberellic acid (GO:0009740) and the cellular response to gibberellin stimulus (GO:0071370) may account for the large differences in phenotypic root growth (average size and root volume) between the SS and BB groups, as gibberellins can reduce root growth, resulting in less root surface area and a smaller average diameter of roots [40]. A study on biochar (a type of OM) added to soil showed that it enhanced tomato shoot growth through the stimulation of the GA pathway, while germination tests, application of exogenous GA, and mutant analysis supported the involvement of the GA pathway in biochar-mediated plant growth promotion [41].

Many of the GO terms of the turquoise module (correlated to BS samples; *p* = 0.87) were involved in cell growth and protein function including deubiquitinase activity (GO:0101005) and DNA metabolic processes (GO:0006259). The highest upregulated genes were involved in a response to salicylic acid (GO:0009751) and circadian rhythm (GO:0042752), both of which may be a response to biological life present in the immediate rhizosphere [42,43]. Deubiquitinase enzymes function to remove ubiquitin from proteins, regulating protein stability and signaling pathways. Their activity in roots could be important for modulating responses to soil nutrient availability or stress [44]. Salicylic acid is a plant hormone involved in the defense response and its increased activity may suggest a priming of the plant’s defense mechanisms, possibly due to the OM influencing the soil microbiome. Circadian rhythms in plants regulate various physiological processes, including growth, hormone signaling, and stress responses. Changes to microbiology within the rhizosphere as a result of adding OM may influence circadian rhythm genes, thus affecting the overall growth and adaptation strategies of the roots in their changing environment [43]. Precise changes due to systemic signaling rather than proximity to soil conditions will require further exploration, but the general exploration of treatment groups allows for better understanding of how clusters of genes are expressed differently according to non-uniform conditions.

**Figure 3:**
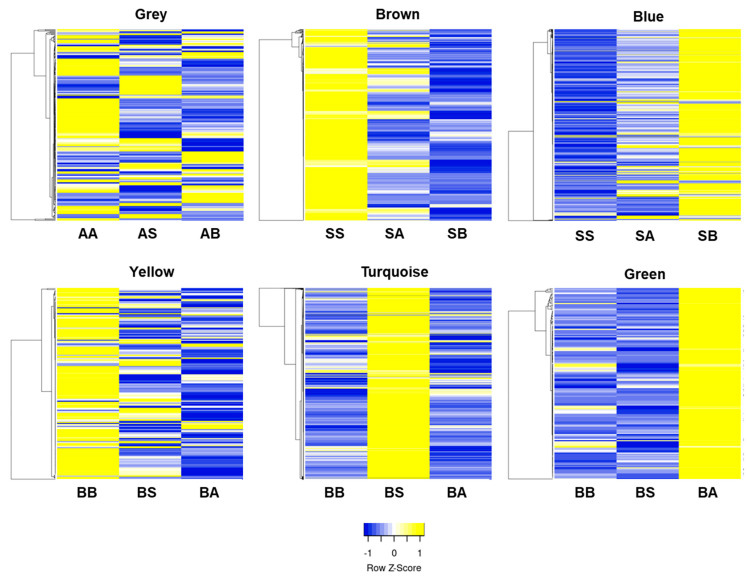
Heatmaps of the genes in the co-expression modules in the soil group where the color module was highly correlated with at least one treatment. Gene expression was indicated by the *z*-score with blue and yellow shades indicating up- and down-regulated genes, respectively.

Heat maps made of each module show that uniform and non-uniform soil treatments did not correlate to similar modules (Figure 3). The S group, which did not have OM or soil life, clustered within either the 364 genes of the brown module or 1286 genes of the blue module, with the blue module revealing genes that are influenced by the non-unform soil conditions when soil life and OM are present on other roots within the same plant. When comparing the A group, which had OM but no soil life, to the 260 genes that clustered within the grey module, samples from both AS and AB showed differences in expression. Similarly, the B group clustered within either the yellow (189 genes), turquoise (1400 genes), or green modules (155 genes), one for each treatment BB, BA, or BS, respectively. The separation of clusters within the B group appears to show how adjacent soil can influence the expression pattern of growing peach roots in a similar environment.

## 4. Conclusions

This is the first exploration of the effect of non-uniform soil conditions resulting from an increase in OM with or without biological soil life on the peach root transcriptome. Peach roots in different soil environments had unique gene expression based on soil type as well as surrounding soil factors, including OM and soil life. More importantly, the differences in DEGs in non-uniform soil conditions show that local root expression can be influenced by soil factors in distant portions of the plant, and the dissimilar modules between the same soil group point to the influence of adjacent soil on the expression of growing peach roots. These initial findings provide opportunities for the future exploration of modules that correspond to root conditions and could help identify how non-homogenous soil conditions may alter plant performance.

## Figures and Tables

**Table 1:** Select nutrients and parameters of the compost used in the experiment.

Nutrient or Characteristic	Value
Total N (g kg^−1^)	8.3
P (g kg^−1^)	2.0
K (g kg^−1^)	2.2
Ca (g kg^−1^)	8.5
Mg (g kg^−1^)	1.4
S (g kg^−1^)	0.08
Zn (mg kg^−1^)	0.04
Cu (mg kg^−1^)	0.02
Mn (mg kg^−1^)	0.16
Fe (mg kg^−1^)	5.14
Na (mg kg^−1^)	0.71
Al (mg kg^−1^)	5.70
C:N (ratio)	11.7
OM (%)	19.4
EC (dS m^−1^)	0.63
pH	7.50

**Table 2:** Soil treatment codes were determined by the soil in which peach roots were collected (first letter) and the adjacent soil environment (second letter) from the three experiment treatments (“sand” as control (S group, without soil life or organic matter (OM)), autoclaved compost (A group, with autoclaved OM), and compost (B group, with biologically active OM)). The adjacent soil resulted in an adjacent soil factor (presence/absence (+/−) of OM and/or soil life between the two contiguous pots).

Treatment Code	Soil Where Root Tissue Was Sampled	Adjacent Soil	Adjacent Soil Factor
SS	S	S	n/a
AA	A	A	n/a
BB	B	B	n/a
SA	S	A	+ OM
SB	S	B	+ soil life and OM
AS	A	S	− OM
AB	A	B	+ soil life
BS	B	S	− soil life and OM
BA	B	A	− soil life

**Table 3:** Peach root length (cm), surface area (area, cm^2^), average diameter (Avg dia, mm), root volume (cm^3^), and number of tips, forks, and crossings by soil type.

Treatment	Length	Area	Avg Dia	Root Volume	Tips	Forks	Crossings
AA	1845.9	458.1	0.8	9.0	3569	6602	1266
BB	711.5	556.1	2.5	34.6	1594	3219	667
SS	1658.4	358.3	0.7	6.2	2978	7882	2259

**Table 4:** Total and down- and upregulated DEGs when comparing peach roots sampled from three uniform and non-uniform soil groups (control (S group, without soil life or organic matter (OM)), autoclaved compost (A group, with autoclaved OM), and compost (B group, with biologically active OM)) and compared by the presence/absence of a specific component (OM and/or soil life) shown by the adjacent soil factor (Log_2_FC > |2|, *p* < 0.01).

Group	Comparison	Adjacent Factor	Total	Down	Up
	SS vs. AA	OM	317	188	129
Uniform	AA vs. BB	soil life	121	53	68
	SS vs. BB	OM and soil life	370	176	194
S	SA vs. SS	OM	200	98	102
− soil life	SA vs. SB	soil life	221	63	158
− OM	SB vs. SS	OM and soil life	411	274	137
A	AS vs. AA	OM	94	52	42
− soil life	AB vs. AA	soil life	83	53	30
+ OM	AS vs. AB	OM and soil life	19	8	11
B	BS vs. BA	OM	332	193	139
+ soil life	BA vs. BB	soil life	106	33	73
+ OM	BS vs. BB	OM and soil life	239	54	185

## Data Availability

The data presented in the study are publicly available and can be found here: https://www.ncbi.nlm.nih.gov/bioproject/PRJNA1019649.

## Supplementary Materials

The following supporting information can be downloaded at: https://www.mdpi.com/article/10.3390/genes15010070/s1.
